# The Effect of the Queen’s Presence on Thermal Behavior and Locomotor Activity of Small Groups of Worker Honey Bees

**DOI:** 10.3390/insects11080464

**Published:** 2020-07-23

**Authors:** Przemysław Grodzicki, Bartosz Piechowicz, Michał Caputa

**Affiliations:** 1 Department of Animal Physiology and Neurobiology, Faculty of Biological and Veterinary Sciences, N. Copernicus University in Toruń, Lwowska 1, 87-100 Toruń, Poland; caputa@umk.pl; 2 Department of Animal Physiology and Reproduction, Faculty of Biotechnology, University of Rzeszów, Werynia 2, 36-100 Kolbuszowa, Poland; bpiechow@poczta.onet.pl

**Keywords:** worker honeybees, the queen, thermal preference rhythm, activity rhythm, clustering, isolation stress

## Abstract

We examined effects of the queen’s presence on diurnal rhythms of temperature preference (TP) and locomotor activity (LA) in worker honeybees’ groups. TP and LA of six queenless and six queenright (with the queen) groups of bees, consisting of 7–8 worker bees, were recorded in a thermal gradient system for four days, under light to darkness (LD) 12:12 photoperiod. The same experiments were conducted on five virgin queens (of the same age as those in the queenright groups), which were placed individually in the gradient chambers. The single virgin queens showed signs of distress and no rhythms of TP and LA. In contrast, there were diurnal rhythms of TP and LA in both group variants with daytime activity and nighttime rest. However, the queen’s presence exerted a strong calming effect, reducing LA of bees both at day- and nighttime. The nighttime minimum LA of queenright groups was five times lower than that in queenless groups. Moreover, there was a reversal of the diurnal pattern of TP in queenright groups. The results are discussed in terms of the bee colony organization as a superorganism.

## 1. Introduction

The honeybee (*Apis mellifera* Linnaeus, 1758) is a species living in societies counting up to 80 thousand individuals [1]. They intercommunicate using sophisticated mechanical and chemical communication systems [2,3,4,5,6]. In a colony, reproduction ability and division of labor are based on morphological and physiological polymorphism, including differences between a prolific queen and the rest of infertile female workers [7,8]. The queen marks her reproductive domination by secreting pheromones. These substances play a crucial role in the honeybee colony organization throughout the entire life of the queen [4,9,10,11,12].

Regarding their thermal biology, single honeybees are heterotherms (i.e., they actively maintain their body temperature higher than ambient temperature or (alternately) they passively follow changes of the latter). During the periods of their motor activity, these insects are endothermic (i.e., they obtain heat for body temperature elevation using their own energy metabolism), while, during resting periods, they become ectothermic (i.e., their body temperature follows changes in ambient temperature) [13,14,15,16,17,18,19,20,21,22,23,24]. Moreover, they rely on social thermoregulation maintaining the in-hive temperature at an almost constant level over the year-round [18,25,26,27,28]. A complex structure and function of the honeybee colony makes it a superorganism analogous to a single, highly organized animal of the same body mass, such as bird or mammal [15,16,29,30,31,32,33]. In honeybees, many physiological and behavioral mechanisms are under the control of social cues synchronizing the colony rhythms [34,35].

Our previous study concerning the analysis of the social behavior of bees in a temperature gradient indicates that small groups of workers, counting about a dozen workers, seem to constitute a simplified but reliable model of the thermal behavior of a bee colony [29]. Smaller groups of workers exhibited thermal behavior similar to that shown in single bees separated from their sisters [29,36,37]. Instead of choosing higher ambient temperature at night (than that during the day), they selected the lowest temperature at night and thus they saved energy, which was likely to be necessary for morning warm up under outdoor conditions. An important question is what makes small groups of bees behave as a colony. One of the integrating factors is the own colony odor emitted by pieces of fresh honeycomb wax [38]. The most important factor, however, should be the presence of the queen and her pheromone impact on the worker honeybees.

In this study, we investigated the effects of the queen’s presence on behavioral thermoregulation and locomotor activity rhythms of worker honeybees. The study aimed to verify a hypothesis that the queen’s presence synchronizes thermal behavior and locomotor activity rhythms of extremely small groups of worker honeybees, making them behave as a colony.

## 2. Materials and Methods

### 2.1. Animals

Three sets of experiments were conducted using: (i) five isolated, six-day-old virgin queens; (ii) six queenless groups of worker honeybees; and (iii) six queenright groups of worker honeybees (*Apis mellifera carnica* Pollman, 1879). Each queenless and queenright group consisted of 8 and 7 workers, respectively. They were nurse bees (aged 6–16 days), creating royal retinues for six unfertilized six-day-old queens. All queens and worker honeybees were obtained from the same apiary. Each set consisting of the queen and her retinue was collected from six different hives. However, the members of the queenless groups were separated from the queens just before placing them into thermal gradient chambers.

### 2.2. Experimental Procedure

We placed the experimental groups into a set of four thermal gradient chambers, running simultaneously. The apparatus (producer: Andrzej Zienkiewicz Zakład Remontowo-Montażowy Aparatury Laboratoryjnej, Toruń, Poland) consisted of four 1 m × 0.08 m × 0.035 m computer-controlled aluminum chambers. One end of each chamber was cooled by a cryostat K21 E20 (GK Sondermaschinenbau GmbH—Labortechnik Medingen), while the opposite end was simultaneously heated by a thermostat, which generated a linear temperature gradient, ranging from 7 to 43 °C. The temperature distribution in each chamber was recorded by a series of 16 thermocouples disposed at equal distances along its floor. The temperature data were processed and recorded by the computer (OS: Windows XP) by means of Grad-K.exe software (programmed by Andrzej Zienkiewicz Zakład Remontowo-Montażowy Aparatury Laboratoryjnej, Toruń, Poland—no numbered versions). The software was a part of the equipment. Bees had ad libitum access to water and 50% sucrose syrup put in four pairs of small dishes distributed at equal distances along the chambers. After 24-h adaptation to the experimental conditions, the animals were subjected to four days of recording of their thermal behavior and locomotor activity. Infrared registration of the insects’ behavior was performed using a Sony Handycam HDR-SR5E video camera. The video recordings were stored on the hard disk of the computer working under the Pinnacle Studio ver.13 software. The experiments were conducted under LD 12:12 photoperiod (light phase 51 lx, white LED lamp, and dark phase 1.5 lx, red LED lamp; the white lights off 18:00–06:00). The registration was done based on a visual inspection of the video films, to locate the insects in the thermal gradient chamber. The location was recorded in the Excel workbook, which contained data concerning temperature distribution in each gradient chamber. That way, the exact value of the ambient temperature selected by each member of each group was determined. The criterion for assessing locomotor activity was whether during each 10-s scene a bee was moving (i.e., walked, run, flew, or changed its position in relation to the closest surrounding) (1) or remained stationary (0). We applied that criterion separately to each insect, and then pooled the obtained data, and averaged them for the entire group over a given period of time, getting the activity index (AI).

A separate series of the experiments was conducted according to the above-mentioned scheme on isolated virgin queens, which were placed individually in the gradient chambers.

### 2.3. Data Analysis

For each experimental series, 15-min averages (±SD) of selected ambient temperature and locomotor activity were calculated. Those averages were used to calculate 24-h, daytime, and nighttime group averages (±SD), as well as those of daily maximum and minimum group values of selected ambient temperature and locomotor activity. Two-hour periods, i.e., eight consecutive 15-min values making the daily maximum and minimum, were selected from each recording and then averaged. We also averaged occurrence times of each maximum and minimum ambient temperature and locomotor activity, which we referred to as the mean time ± SD values.

Selected ambient temperature and locomotor activity were also examined in the context of the queens’ and worker bees’ circadian rhythmicity. We compared the period, acrophase, amplitude, and average values of the rhythms in single queens and in the queenless and queenright groups of worker bees using the software Acro and Cosinor [39].

The statistical significance of differences in selected ambient temperature and in locomotor activity between queenless and queenright groups of worker bees in each of two phases of the circadian rhythm and the significance of the effect of the queen’s presence was determined using two-way ANOVA with the time of day as the repetitive variable. Each analysis was followed by Tuckey’s post hoc test for unequal N (Spjotvoll/Stoline test). In the case of single queens, daytime and nighttime averages were compared using Student’s paired *t*-test.

During the data analysis, we assumed that, in terms of thermal preference and locomotor activity, each honeybee group behaved as the unity representing a simplified model of honeybee colony [29]. Therefore, in the analysis, we treated them as separate units (*n* = 6 means six groups, not six animals). Diagrams shown in the Results Section represent the averaged behavior of all individuals composing the group.

## 3. Results

In general, thermal preference of single queens was not affected by the time of day (Student’s paired *t*-test daytime vs. nighttime; *p* = 0.2899), while there was a significant difference in locomotor activity between daytime and nighttime (Student’s paired *t*-test; *p* = 0.0372). As far as groups of worker bees are concerned, the time of day (daytime vs. nighttime; two-way ANOVA F_1,10_ = 0.31; *p* = 0.5922) and the queen’s presence in the group (two-way ANOVA F_1,10_ = 0.18; *p* = 0.6832) had no effect on the bees’ temperature preference but strongly affected their locomotor activity (two-way ANOVA, time of the day effect F_1,10_ = 514.54; *p* = 0.0000; the queen presence effect F_1,10_ = 51.65; *p* = 0.00003). However, the presence of the queen modified the daytime and nighttime averages of ambient temperature selected by bees (two-way ANOVA, the interaction F_1,10_ = 13.76; *p* = 0.0040). On the other hand, there was no such an alteration in locomotor activity (two-way ANOVA, interaction F_1,10_ = 3,51; *p* = 0.0903).

### 3.1. Thermal Preferences

**Figure 1:**
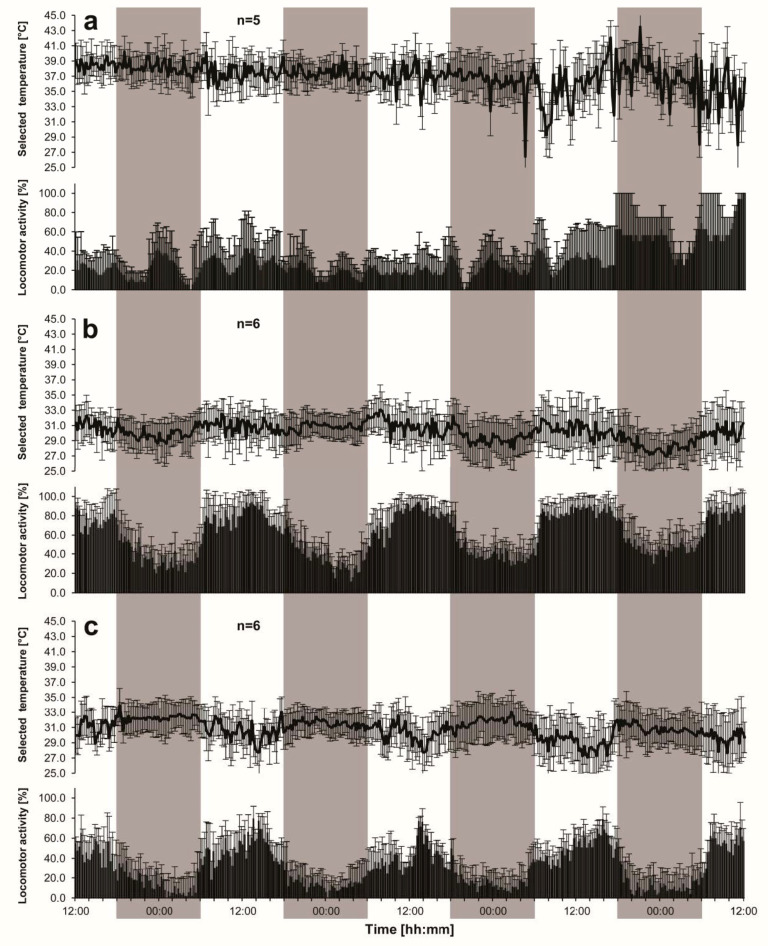
Daily changes in mean (±SD) values of thermal preference and locomotor activity of single queens (**a**) and queenless (**b**) as well as queenright (**c**) groups of worker honeybees.

Single queens did not show any visible circadian oscillations of thermal preference during the initial three days (Figure 1a). At the beginning of the fourth day, however, they tended to select lower a temperature than at the start of the next night.

In both queenless and queenright groups, smoothed courses of the 15-min averages would have clear-cut shapes of the sinusoid waves (Figure 1b,c). In queenless groups (Figure 1b), their zeniths occurred at every daytime period (at 13:52 ± 01:55, 11:43 ± 01:20, and 14:31 ± 01:41, during the final three days, respectively), while nadirs were always recorded at night (at 00:50 ± 01:46, 02:34 ± 02:40, and 02:09 ± 01:20, respectively). In queenright groups (Figure 1c), the circadian rhythm was characterized by a phase-shift by about 12 h. Over the final three days of the recording the single (15-min averages) minimum values occurred at daytime (at 16:50 ± 01:20, 12:33 ± 01:20, and 17:31 ± 01:41, respectively), and the maximum values were recorded at night (at 20:40 ± 01:46, 21:43 ± 02:11, and 21:31 ± 01:13, respectively).

**Figure 2:**
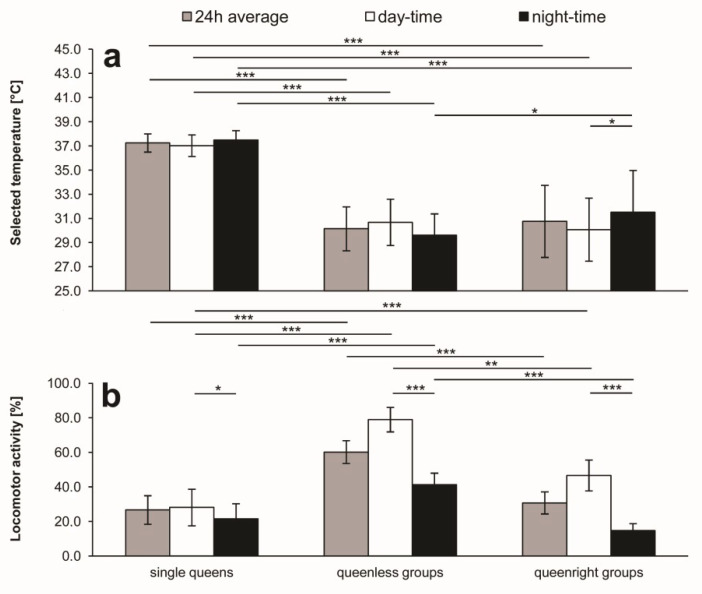
The 24-h, daytime, and nighttime averages (±SD) of ambient temperature preference (**a**) and locomotor activity (**b**) of single queens and queenless as well as queenright groups of worker honeybees. Asterisks indicate the statistical significance of differences between means: * *p* < 0.05, ** *p* < 0.01, *** *p* < 0.001.

Figure 2a shows that there were no statistically significant day–night differences in thermal preference of single queens or in that of queenless groups of worker bees, while the bees in queenright groups preferred significantly (*p* < 0.05) higher temperatures at night than those during the day. In queenright groups, the 24-h average of 31.0 ± 3.0 °C and the daytime average of 30.3 ± 2.6 °C did not differ significantly from those in queenless groups (30.3 ± 2.0 and 30.9 ± 2.0 °C, respectively). On the other hand, the nighttime ambient temperature selected by bees in queenright groups (31.5 ± 3.5 °C) was significantly (*p* < 0.05) higher than that in queenless bees (29.6 ± 1.9 °C). It must be stressed that each of the three averages of thermal preference of single queens (Figure 2a) had a value of 37 °C or slightly more and they were significantly (*p* < 0.001) higher than their counterparts of both queenless and queenright groups of bees. On the other hand, the queens tended to select slightly higher ambient temperature at night than that during the day, but the difference (as mentioned above) was far from being statistically significant.

**Figure 3:**
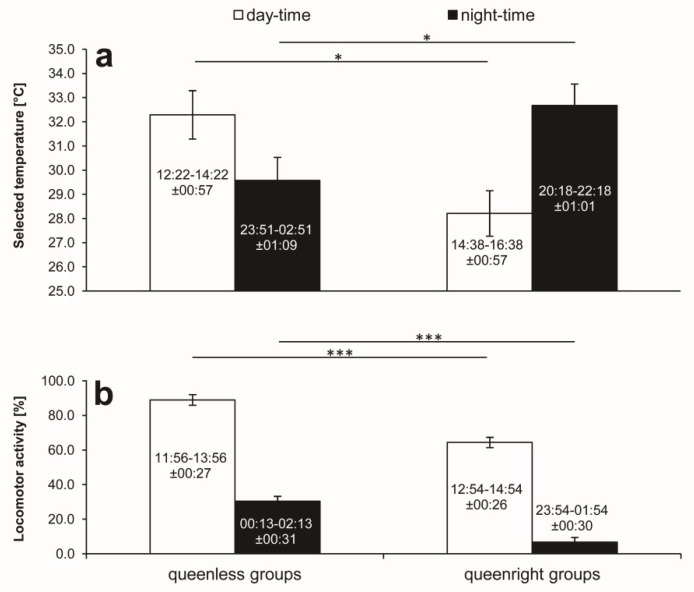
Average values (±SD) of two-hour periods of daily maximum and minimum selected ambient temperature (**a**) and locomotor activity (**b**), as well as the times (in hours and minutes) of their occurrence (±SD) in queenless and queenright groups of worker honeybees. Asterisks indicate the statistical significance of differences between means: * *p* < 0.05, ** *p* < 0.01, *** *p* < 0.001.

Because of the above-mentioned clear-cut daily shifts in thermal preferences of worker bees’ groups (illustrated by Figure 1), we decided to compare all individual groups’ two-hour daily maxima and minima of the preference. This extra analysis, presented in Figure 3a, shows that the queen’s presence did not affect the daily extremes significantly (two-way ANOVA F_1,18_ = 0.143; *p* = 0.715).

On the other hand, an interaction between the queen’s presence and the time, when the extremes appeared, indicated that the time of the maximum and minimum ambient temperatures selected by bees differed significantly between the queenless and queenright groups (two-factor interaction: F_1,18_ = 10.895; *p* = 0.002). In queenright groups, the minimum temperatures were selected afternoons and the maximum values were recorded at night, which was exactly opposite to the diurnal shifts recorded in queenless groups. The post hoc test demonstrated that the queen’s presence affected both the daytime (*p* < 0.05) and nighttime (*p* < 0.05) extremes of ambient temperatures selected by bees.

### 3.2. Locomotor Activity

Single queens (Figure 1a), similar to both queenless (Figure 1b) and queenright (Figure 1c) groups of the workers, tended to be more active during the day than at night (although the queens exhibited some signs of excitement during the last night). The difference was significant, as shown by Student’s paired *t*-test (*p* < 0.05) (Figure 2b). In the group experiments, the diurnal course of the activity index (AI) oscillated parallel to that of selected ambient temperature in queenless groups (Figure 1b, Figure 2 and Figure 3), while, in queenright groups, the courses were opposite to each other (Figure 1c, Figure 2 and Figure 3). Daily oscillations of locomotor activity of the workers (Figure 1, Figure 2 and Figure 3) were under a clear influence of the queen’s presence (two-way ANOVA F_1,10_ = 51.62; *p* = 0.00003), and of time of the day (averages for daytime vs. nighttime; two-way ANOVA F_1,10_ = 513.87; *p* = 0.0000). On the other hand, the queen’s presence did not modify the effect of time of the day (two-way ANOVA, interaction F_1,10_ = 3.52; *p* = 0.0899). In queenless groups, the 24-h average of AI was as high as 60.2 ± 6.6% and its daytime and nighttime values were 79.0 ± 7.1% and 41.3 ± 6.7%, respectively (Figure 2b). The day–night difference was highly significant (*p* < 0.001). In queenright groups, the 24-h average of AI (30.7 ± 6.4%) was reduced to a half of that recorded in queenless groups (*p* < 0.001). Its nighttime value of 14.7 ± 4.0% was significantly lower than the daytime value of 46.7 ± 9.0% (*p* < 0.001).

Figure 3b shows that the queen’s presence significantly reduced both daily two-hours maxima and minima of locomotor activity of the workers’ groups (F_1,19_ = 44.382; *p* = 0.000). The post hoc test showed that both the maxima and minima in the queenright groups were highly significantly lower (*p* < 0.001) than those in the queenless groups. In both experimental groups, the maximum activity values were recorded early afternoons, while the minimum values appeared just after midnight, and there were highly significant differences between the extremes (F_1, 19_ = 828.539; *p* = 0.000). However, there was no interaction between the queen’s presence and the times of the extremes (F_1,19_ = 0.057; *p* = 0.813).

### 3.3. Clustering Behavior

**Figure 4:**
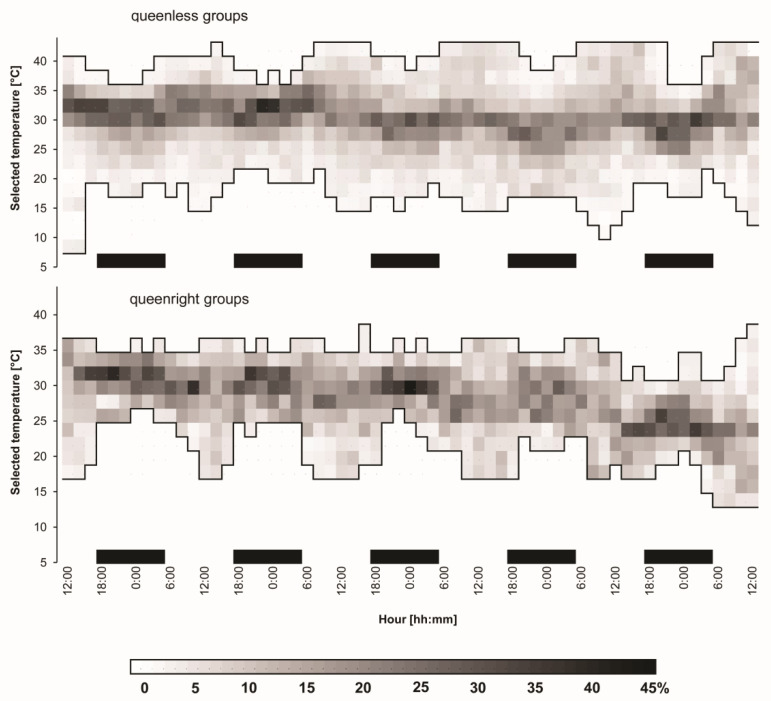
A diagram illustrating the dynamics of worker bees’ clustering behavior in queenless and queenright groups of the bees over four-day recording period. The small squares filled with different grades of grey indicate percentage of the groups’ members clustering together at particular temperature points of the gradient chambers. The percentage scale of the clustering is presented at the bottom of the figure.

Both queenless and queenright groups of worker bees clustered every night and dispersed every day (Figure 4). However, groups accompanying the queen generally occupied much narrower areas of the gradient chambers than those penetrated by queenless groups. In queenright groups, the span between the coldest and the warmest worker localization in the gradient reached about 25 °C during the day and was reduced to as little as 10 °C at night, while in queenless groups the respective values were 40 and 20 °C.

During the day, bees of queenless groups penetrated much warmer areas of the gradient chambers (up to 43 °C) than did bees of queenright groups (usually below 37 °C). At night, the respective minimum temperatures were 17–22 and 22–27 °C. The figure shows clearly that bees in queenright groups each night escaped from cooler areas of the gradient chambers to aggregate at higher temperatures.

### 3.4. Relationship of Circadian Rhythms of Selected Ambient Temperature and Locomotor Activity

As mentioned above, the courses of temperature preference and locomotor activity of worker bees’ groups took the form of a sinusoidal curve, indicating that these variables oscillate in a circadian rhythm. Therefore, it was possible to fit the curve to assess the mean value, period, acrophase, and amplitude of the rhythm. The results are shown in Table 1. The average period of the rhythm of selected ambient temperature was 24.6 ± 0.6 h for queenless and 24.2 ± 0.8 h for queenright groups. The difference was insignificant. For both queenright and queenless groups, the period of the locomotor activity rhythm was 23.8 ± 0.2 h. The slight difference between periods of rhythms of the selected ambient temperature and locomotor activity was also statistically insignificant.

## 4. Discussion

In this study, we examined the impact of the queen’s presence on thermal behavior of extremely small groups of worker honeybees. The queen’s presence resulted in a clear-cut modification of thermal preference, locomotor activity, and clustering behavior of the groups.

Similar to projects described in our previous papers [29,36], we used small groups of worker honeybees. In the studies, we recorded a tendency to reverse the circadian rhythm of thermal preference of the queenless groups of bees containing no more than 20 members in comparison to that of single bees separated from their sisters. The isolated bees selected significantly higher ambient temperature during the day than those at night, which was confirmed in our subsequent studies [37,38]. In another investigation (unpublished data), we examined the effect of group size and found the tendency to reverse the day–night shifts in selected ambient temperature at the size exceeding seven worker bees in queenless groups. In the present investigation, we also used such extremely small queenless and queenright groups of worker bees containing eight and seven workers, respectively. Queenless groups exhibited thermal behavior similar to that previously evidenced in single bees separated from their sisters [29,36,37]. However, in this study, there was a clear-cut reversal of the circadian pattern of thermal preference in queenright groups, i.e., they selected higher ambient temperatures at night than those during the day. On the other hand, there was no reversal in the pattern of their locomotor activity. Both queenless and queenright groups were significantly more active during the day than at night. However, the queen’s presence exerted a calming effect, i.e., it strongly reduced activity of bees both at daytime and nighttime. The effect was the strongest during two-hour periods of the nighttime minimum activity, when queenright groups were as much as five times less active than their queenless counterparts (see Figure 3). This suggests that the queen’s presence might promote a sleep-like state in bees at nighttime. In our previous investigation [38], we showed that the own colony odor, emitted by fresh beeswax pieces placed in the gradient chambers, also calmed isolated bee workers, but the effect was not so strong and the odor did not modify the daily pattern of the bees’ thermal preference, i.e., they kept on selecting higher ambient temperatures during the day than those at night. Such a pattern, which is typical for single worker bees separated from their sisters, represents a heterothermic strategy of nocturnal saving energy necessary for morning warm-up under outdoor conditions [29]. The only difference between the extremely small queenless groups of worker bees in the present investigation and those recorded previously in isolated bees concerns the day–night amplitude of thermal preference, being much bigger in the latter. On the other hand, the reversal of the pattern in queenright groups represents a homeothermic strategy characterized by an increase in selected ambient temperature to compensate for a reduction in muscular heat production during the nocturnal inactivity of worker bees. The 12-h phase shift in the small queenright groups, comparing to the rhythm of small queenless groups, is then a clear-cut quantitative change making the former behave as a swarm.

Bees accompanying the queen clustered more closely than those in queenless groups. The effect of the queen’s presence strengthened each night, resulting in clustering of worker bees at higher ambient temperatures (see Figure 4).

The experiments performed on isolated virgin queens showed that they tended to select slightly higher ambient temperatures at night than during the day (see Figure 2a), but the difference was far from being significant. On the other hand, the queens were significantly more active during the day than at night (see Figure 2b). Nevertheless, the acro and cosinor analysis showed no rhythm of both the queens’ thermal preference and their locomotor activity. The latter is incompatible with the finding of Harano, Sasaki, and Sasaki [40], who showed moderate synchronization of locomotor activity to light phase in isolated virgin queens. The only difference in the treatment of the queens by the Japanese investigators, comparing to that in the present study, concerns supplementation of their queens with royal-jelly solution. Therefore, we cannot exclude that the lack of the rhythm in our queens was due to the distress induced by this alimentary deprivation. An unexpectedly high ambient temperature of ~37 °C, selected by our queens (see Figure 1a and Figure 2a), as well as clear signs of excitement visible during the last night of the recording (see Figure 1a), can be regarded as an indirect confirmation of their emotional distress. Such a temporary increase in selected ambient temperature is called behavioral fever, which has been described in various classes of both vertebrates and invertebrates. To the best of our knowledge, there are no published data concerning thermal behavior of the queens. The high ambient temperature was selected by our queens both during the day and at night, which is quite different from the heterothermic strategy used by single isolated worker bees [29,36,37]. Clearly, there are mutual stabilizing effects of chemical signals emitted by the queen and received by the workers and those provided by the workers (honey and royal-jelly) and received by the queen.

The queen affects worker bees’ behavior with volatile chemical signals. The quantitative composition of chemical substances secreted by the queen changes with her age and physiological state [4,9]. In the honeybee colony being under full control of the queen, worker bees adjust their physiological and behavioral rhythms to those of the queen [9,34,41,42].

Members of the colony show endogenous physiological and behavioral rhythms. The rhythms are conditioned, besides the above-mentioned influence of the queen, by various social signals originated from other workers, drones, and offspring [43]. The circadian rhythms of thermal preference and locomotor activity, manifested by both queenless and queenright groups, could be due to the social signals and environmental cues such as photoperiod, season of the year and weather conditions. All these factors synergize with each other.

We conducted our experiments in the late spring and summer. In these seasons, honeybee colonies are subjected to many structural changes, such as an increase in the number of worker bees, occurrence of swarming mood, and the colony division. These changes, as well as the colonization of a new beehive, growth and development of the daughter colonies, and storage of backup materials for wintering are also induced by specific chemical signals [1,44]. In summer and autumn, small groups of queenless worker bees progressively tended to lose their diurnal rhythm of thermal preference [36]. In this context, the queen’s presence, in the present investigation, can be regarded as a factor fixing the rhythm reversal in worker bees groups.

In summary, the queen’s presence exerts profound effects on worker honeybees accompanying her. There was a significant reduction in locomotor activity and the phase shift of the circadian rhythm of ambient temperature selection in queenright groups in comparison to those in queenless groups. All this evidences a close interaction between the queen and the workers, which is probably due to chemical substances secreted by the queen. The effect is stronger than that of the own colony odor. Both are important social stimuli stabilizing the colony. The presence of the brood in the colony seems to be another important factor influencing behavior of worker bees. The offspring need continuous care [45]. This is why the youngest worker bees show intermittent around-the-clock activity. In the offspring absence, our worker bees, in both queenless and queenright groups, showed a clear-cut synchronization of their thermal behavior and locomotor activity with the light phase.

## 5. Conclusions

The queen’s presence results in a clear-cut modification of thermal preference, locomotor activity, and clustering behavior of worker honeybees. The queen’s presence reverses the rhythm of ambient temperature selected by small worker honeybee groups, changing their thermal behavior from the pattern typical of single individuals to that of a honeybee colony.

The queen’s presence decreases locomotor activity of the worker honeybees but it does not affect their rhythm of the activity.

Virgin queens’ isolation from their retinues reduces the amplitude of their rhythms of thermal preference and locomotor activity. The isolation also induces behavioral fever symptoms.

## Figures and Tables

**Table 1:** A comparison of the period, acrophase, mean, and amplitude of the rhythms of the selected ambient temperature and locomotor activity in single queens and in queenless and queenright groups of worker bees.

	Selected Temperature				Locomotor Activity			
	Single Queens	Queenless Groups		Queenright Groups	Single Queens	Queenless Groups		Queenright Groups
Period	No rhythm	24.6 ± 1.5 h	ns	24.2 ± 2.0 h	No rhythm	23.8 ± 0.1 h	ns	23.8 ± 0.1 h
Acrophase	No rhythm	11:56 h	*p* < 0.001	00:15 h	No rhythm	13:06 h	ns	13:01 h
Mean	No rhythm	31.0 ± 1.3 °C	ns	30.8 ± 3.0 °C	No rhythm	60.1 ± 2.7%	*p* < 0.001	30.7 ± 2.6%
Amplitude	No rhythm	4.5 ± 0.7 °C	ns	5.2 ± 1.2 °C	No rhythm	44.9 ± 0.7%	*p* < 0.05	46.7 ± 0.8%
